# Comparison between the effects of elective nodal irradiation and involved‐field irradiation on long‐term survival in thoracic esophageal squamous cell carcinoma patients: A prospective, multicenter, randomized, controlled study in China

**DOI:** 10.1002/cam4.3409

**Published:** 2020-08-25

**Authors:** Jiahua Lyu, Abulimiti Yisikandaer, Tao Li, Xiaozhi Zhang, Xiaohu Wang, Zhongge Tian, Long Chen, Bing Lu, Hong Chen, Jie Yang, Qifeng Wang, Jinnrong Zhang, Youguo Ma, Rui Liu, Ruifeng Liu, Aiguri Hage, Jinyi Lang

**Affiliations:** ^1^ School of Medicine University of Electronic Science and Technology of China Chengdu China; ^2^ Sichuan Cancer Hospital Institute Sichuan Cancer Center School of Medicine University of Electronic Science and Technology of China Chengdu China; ^3^ The Affiliated Tumor Hospital of Xinjiang Medical University Xinjiang China; ^4^ The First Affiliated Hospital of Xi'an Jiaotong University Xi'an China; ^5^ Gansu Cancer Hospital Lanzhou China; ^6^ Wu Wei Tumor Hospital Wu Wei China; ^7^ The Affiliated Cancer Hospital of Guangxi Medical University Guangxi China; ^8^ Guizhou Cancer Hospital Guiyang China; ^9^ Kunming General Hospital of Chengdu Military Region Kunming China; ^10^ Xinjiang Renmin Hospital Xinjiang China

**Keywords:** elective nodal irradiation, esophageal cancer, involved‐field irradiation, survival

## Abstract

**Background:**

This study's initial results revealed significant decreases in treatment‐related esophagitis and pneumonitis cases in patients with thoracic esophageal squamous cell carcinoma (ESCC) treated with involved‐field irradiation (IFI), compared to elective nodal irradiation (ENI). This report outlines the long‐term trial results, specifically; overall survival (OS), progression‐free survival (PFS), metastasis‐free survival (MFS), and locoregional progression‐free survival (LRFS).

**Materials and Methods:**

Stage II–III thoracic ESCC patients were assigned randomly, in a 1:1 ratio, into either the ENI or IFI arm. Radiation therapy was delivered once a day in 1.8‐2.0 Gy fractions to a total dose of 60.0‐66.0 Gy to the gross tumor volume and 50.0‐54.0 Gy to the clinical target volume. The primary endpoints were acute treatment‐related esophagitis and pneumonitis. The results for the primary endpoints were previously published in 2018. In this article, we analyzed the secondary endpoints including PFS, LRFS, MFS, and OS.

**Results:**

Between April 2012 and October 2016, 228 patients from nine participating centers in China were enrolled into this study and randomly assigned to two treatment groups. For ENI and IFI groups, respectively, the results showed similarity and were as follows: median PFS (20.3 months vs 21.4 months), OS (32.5 months vs 34.9 months), MFS (28.2 months vs 26.0 months), and LRFS (25.0 months vs 26.6 months). In particular, respective OS rates in the ENI and IFI groups were 84.6% and 82.5% after 1 year, 45.1% and 48.7% after 3 years, and 29.8% and 30.7% at 5 years. PFS rates after 1, 3, and 5 years were 58.9%, 34.2%, and 26.9%, respectively, in the ENI arm compared to 64.4%, 30.8%, and 27.7%, respectively, in the IFI arm. Multivariate analysis identified clinical stage and tumor responses as independent predictors of OS. Meanwhile, tumor location, cStage, and tumor response were identified as independent factors influencing PFS.

**Conclusion:**

IFI was associated with similar survival as ENI in patients with thoracic ESCC, suggesting that IFI is an acceptable treatment method for thoracic ESCC.

## Graphical abstract

The results of this study illustrated that IFI was associated with similar progression‐free survival (PFS), locoregional progression‐free survival (LRFS), metastasis‐free survival (MFS), and overall survival (OS) as ENI in patients with clinically inoperable thoracic esophageal squamous cell carcinoma (ESCC). In multivariate analysis, clinical stage and tumor response were identified as independent predictors of overall survival, whereas tumor location, cStage, and tumor response were predictive of progression‐free survival.

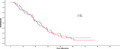

## 1 INTRODUCTION

In terms of worldwide cancer‐related death causation, esophageal carcinoma (EC) is sixth and ninth for men and women, respectively.(1) In China, EC was the third most common cancer in 2015, with an estimated 477 900 new cases reported, and the fourth most common cause of cancer deaths, estimated at 375 000.(2) Patients who decline surgery or have unresectable EC are given concurrent chemoradiotherapy (CCRT) as standard therapy.(3)

In radiotherapy, optimal delineation of the target volume is critical for improving therapeutic efficacy and reducing chemoradiation‐induced toxicity. However, a consensus is lacking regarding the optimal radiation field design for nodal irradiation. Elective nodal irradiation (ENI) has been recommended as the initial irradiation strategy for patients with EC. However, ENI often induces higher incidences of treatment‐related complications because of the larger radiation volume. Theoretically, involved‐field irradiation (IFI), with its smaller target volume, should reduce radiotoxicity compared with ENI. This has led to reevaluation of IFI's role in EC treatment in recent years.

However, the smaller clinical target volume (CTV) range of IFI has been suggested to decrease tumor control and patient survival. Although a few studies confirmed similarity of locoregional control, overall survival (OS), and progression‐free survival (PFS) between ENI and IFI, and that IFI may be accompanied by reduced rates of toxicity,(4, 5) there is no direct evidence from high‐quality randomized clinical trials.

Therefore, we assessed the clinical outcomes and toxicities in EC patients definitively undergoing ENI or IFI with concurrent chemotherapy through this multicenter, controlled, randomized, prospective study. Our initial results were published in 2018.(6) We found that IFI was associated with lower incidences of grade 2 or worse treatment‐related esophagitis (34.7% vs 19.2%, *P* = .018) and pneumonitis (8.7% vs 18.8%, *P* = .027) than ENI in patients with thoracic esophageal squamous cell carcinoma (ESCC).

As the toxicity benefit in IFI is not being questioned anymore, a special attention has been directed towards whether IFI would have a long‐term survival comparable to ENI. This encouraged us to run a second wave of the study. In this article, we analyzed and reported our study's secondary endpoints, including OS, metastasis‐free survival (MFS), locoregional progression‐free survival (LRFS), and PFS.

## 2 MATERIALS AND METHODS

### 2.1 Patient study inclusion criteria

We recruited patients to the study and assessed their eligibility based on the following conditions: (1) inoperable, histologically verified, Stage II–III thoracic ESCC; (2) Karnofsky performance status (KPS) ≥70; (3) age of 18‐75 years; (4) adequate hematological, renal, hepatic, and pulmonary function (defined as a platelets ≥100 000 cells/mm^3^, neutrophils ≥1500 cells/mm^3^, hemoglobin ≥9.0 g/dL, serum creatinine ≤2.0 mg/dL, bilirubin ≤ 1.5 × the normal upper level at the institution and transaminase ≤ 3 × the normal upper level). Being pregnant, breast‐feeding, having an active second carcinoma, metastasis, or serious cardiovascular disease (having a pacemaker) were all criteria for study exclusion. Additionally, patients were excluded if they had tumors invading into the bronchi or trachea or showed the presence of a tracheoesophageal fistula.

Written informed consent was obtained from all participants. Our institution's Ethics Committee reviewed the protocol for accordance with Declaration of Helsinki (1964) and all research experiments undertaken complied with its tenets (ethics number, SCCHEC2012008). The ClinicalTrials.gov study identifier of this prospective trial is NCT01551589.

### 2.2 Randomization

This was a prospective, randomized, multicenter, controlled study. Patients were centrally randomized by 1:1 assignment using computer‐generated randomization lists to either an IFI or ENI arm.

### 2.3 Radiotherapy

Supine computed tomography (CT) was performed on all patients. 3.0‐mm‐thick CT images were obtained covering the whole thorax. We defined planning target volume (PTV), gross tumor volume (GTV), and tumor CTV (CTVt) for both ENI and IFI identically.

The GTV included the primary cancer (total GTV [GTVt]) and metastatic lymph nodes (nodal GTV [GTVn]). The definition of GTV was a primary tumor detected by barium esophagography, endoscopy, or CT. GTV also included positive [18F]‐fluorodeoxyglucose positron emission tomography (except for physiological build‐up) and all lymph nodes with short axis diameters greater or equal to 1.0 cm.

Both Nodal CTV (CTVn) and CTVt were used to establish CTV. CTVt definition encompassed the area of GTVt in addition to 0.8‐1.0 cm on the left and right as well as 3.0 cm above and below the primary tumor.

The CTVn for IFI included the nodal region(s) in which the metastatic lymph node(s) was/were located. Lymph node stations were numbered based on AJCC/UICC Staging: Esophagus and Esophagogastric Junction, 7th Edition. For example, there is a middle thoracic esophageal squamous cell carcinoma patient (T3N1M0) with station 4 lymph nodes (+). IFI CTVn only includes the area of lymph nodes station 4.

ENI CTVn encompassed both clinically involved or uninvolved lymph node regions or stations, respectively, in accordance with the site of the primary tumor (lower, middle, and upper thoracic ESCC: station numbers for lymph node being 4/5/7/8/9/16/17, 2/4/5/7/8/9, and 1/2/4/5/7, respectively).

1.0 and 5 mm longitudinal and radial margins, respectively, were applied to the CTV to generate PTV according to NCCN guidelines and Guidelines for radiotherapy of esophageal cancer in China.

6.0‐MV photons were used to deliver radiotherapy. The dose–volume histogram was used to optimize plans using the following criteria: (1) 95.0% of the PTV was covered by isodose prescription curves and (2) 110.0% of the prescribed dose was the highest upper limit of the PTV dose. Vulnerable organs were limited to the following doses: average heart dose, ≤30.0 Gy, average lung dose, ≤15.0 Gy; V05, V20, and V30, ≤60.0, ≤30.0, and ≤20.0%, respectively, and maximum spinal cord dose, ≤45.0 Gy.

Image‐guided radiation therapy was delivered once a day in fractions between 1.8‐2.0 Gy with a dose summing up to 60.0‐66.0 Gy to the GTV and 50.0‐54.0 Gy to the CTV.

### 2.4 Chemotherapy

Patients received chemotherapy and radiotherapy concurrently. Chemotherapy consisted of 2‐4 cycles of docetaxel (first day dose = 75.0 mg/m^2^) combined with cisplatin (doses on days 1‐3 = 25.0 mg/m^2^) every 21‐28 days. After CCRT, additional 1‐2 rounds of ancillary chemotherapy were given to patients with adequate bone marrow function and a good performance status.

### 2.5 Follow‐up

After patients completed radiotherapy, the Response Evaluation Criteria in Solid Tumors (version 1.1) was used to evaluate tumor reduction. In the first 2 years, follow‐up of patients was conducted every 3 months, then twice in the third year (after each 6 months) followed by once a year until the end of the study.

### 2.6 Endpoints

Primary endpoints were grade ≥2 acute treatment‐related esophagitis and grade ≥2 acute treatment‐related pneumonitis. The primary endpoint results were published in 2018 in *Chinese Journal of Radiation Oncology*. The secondary endpoints included PFS, LRFS, MFS, and OS.

### 2.7 Statistical analyses

Baseline traits between the two treatment groups were compared using the chi‐squared test. The log‐rank test was used to compare Kaplan–Meier curves of OS, PFS, LRFS and MFS. Version 19 of the Statistical Package for Social Sciences (SPSS Inc, IL, USA) was used on a Windows platform to carry out all statistical measurements. The effects of various elements on survival were examined with the Cox proportional hazards model by carrying our multivariate or univariate analyses as required. Elements compared included age (above and below 60 years), sex, treatment group (ENI vs IFI), tumor site, length of tumor (≤5 cm vs >5 cm), KPS score (70‐80 vs ≥90), cStage (II vs III), and response of tumors (partial + complete response vs progressive + stable disease). *P*‐values (two‐tailed) <.05 were taken to indicate statistical significance.

## 3 RESULTS

### 3.1 Characteristics of patients

Two hundred and twenty‐eight patients from nine Chinese study centers agreed to participate after meeting the eligibility criteria from April 2012 to October 2016. One hundred and fourteen eligible patients were randomized to each of the IFI or ENI groups. Sixteen IFI and 20 ENI patients were excluded after they withdrew consent or were lost to follow‐up. Finally, 98 IFI and 94 ENI patients were included in the final analysis. Table 1 shows that there was homogeneity of baseline characteristics between the two groups.

**Table 1:** Patient and tumor characteristics

Characteristic	ENI, n = 94	IFI, n = 98	χ^2^	*P*‐value
Sex, n (%)				
M	71 (75.5)	71 (72.4)	0.237	.742
F	23 (24.5)	27 (27.6)		
Age (years), n (%)				
≥60	44 (46.8)	54 (55.1)	1.321	.312
<60	50 (53.2)	44 (44.9)		
KPS, n (%)				
≥90	44 (46.8)	33 (33.7)	3.446	.077
70‐80	50 (53.2)	65 (66.3)		
Tumor length (cm), n (%)				
≤5	57 (60.6)	60 (61.2)	0.007	.000
>5	37 (39.4)	38 (38.8)		
Location, n (%)				
Ut	37 (39.4)	42 (42.9)	0.300	.861
Mt	49 (52.1)	49 (50.0)		
Lt	8 (8.5)	7 (7.1)		
cStage, n (%)				
II	22 (23.4)	27 (27.6)	0.434	.620
III	72 (76.6)	71 (72.4)		

Note

Abbreviations: cStage, clinical stage; ECOG, Eastern Cooperative Oncology Group; ENI, elective nodal irradiation; F, female; IFI, involved‐field irradiation; KPS, Karnofsky Performance Status scores; Lt lower thoracic; M, male; Mt middle thoracic; Ut upper thoracic.

### 3.2 Survival

Follow‐up for all patients ranged between 4.0 and 81.3 months and lasted for a median length of 40.4 months. The respective median PFS and OS for the whole cohort were 21.0 and 33.8 months.

**Figure 1:**
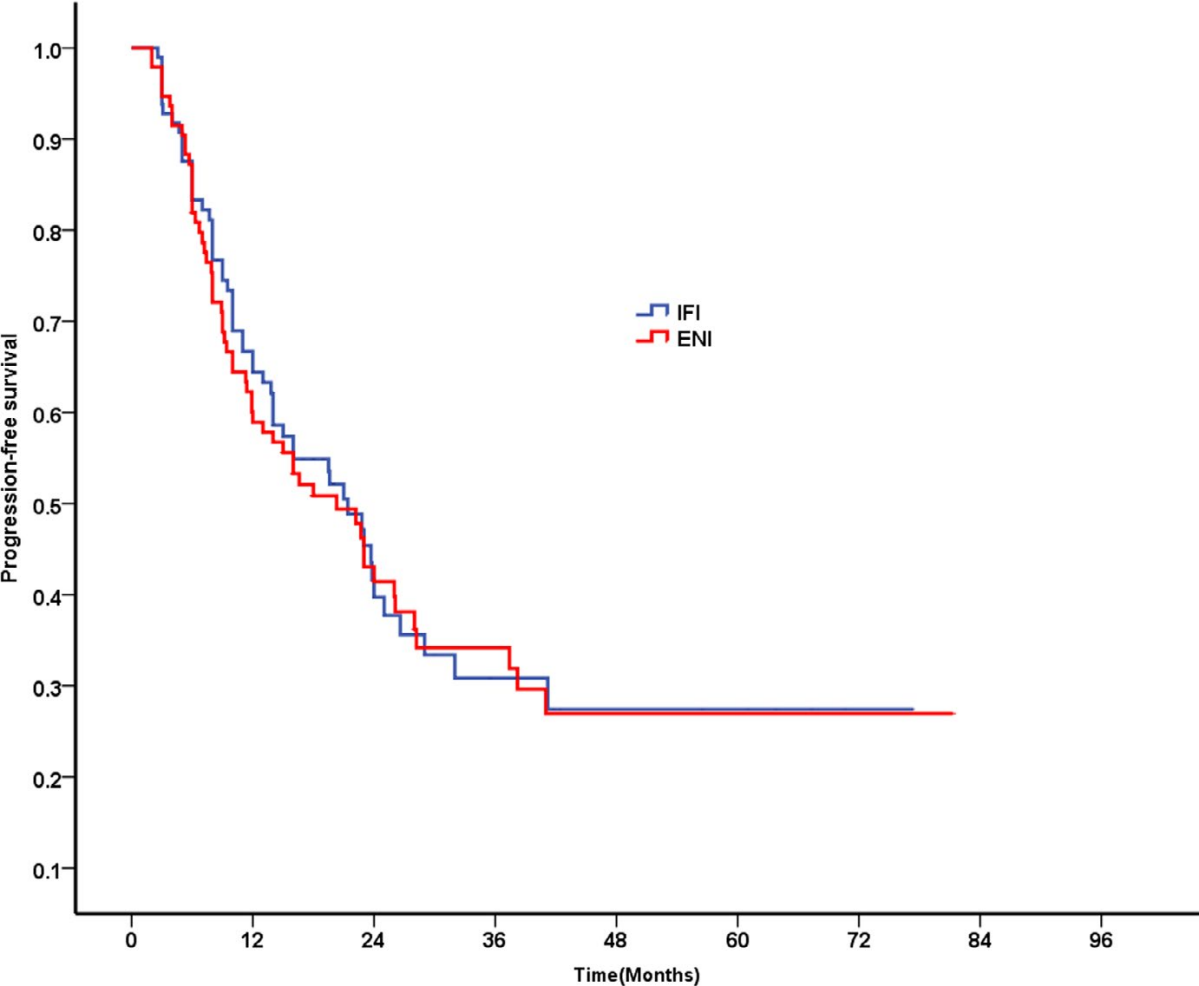
Progression‐free survival for patients in IFI group vs ENI group

**Figure 2:**
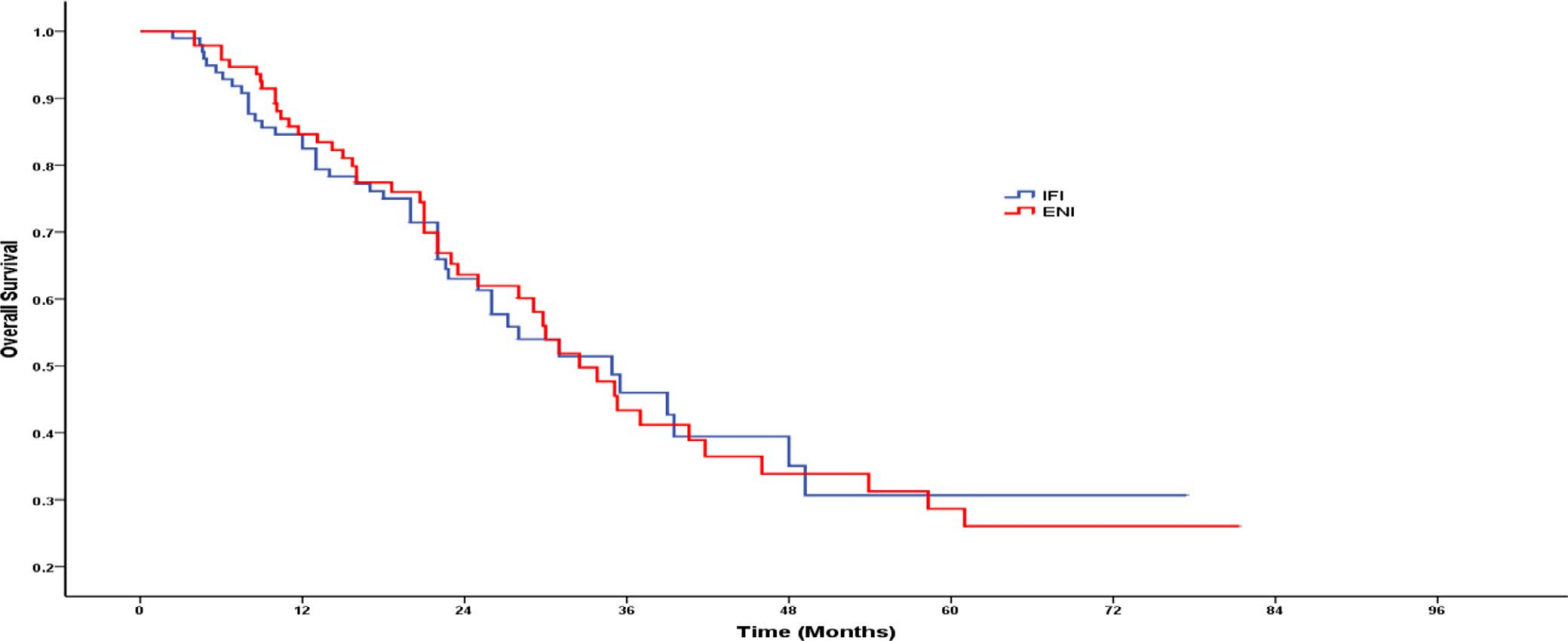
Overall survival for patients in IFI group vs ENI group

The median durations compared between ENI and IFI groups showed similarity and these were PFS (20.3 months vs 21.4 months; *P* = .809), OS (32.5 months vs 34.9 months; *P* = .941), MFS (28.2 months vs 26.0 months; *P* = .983), and LRFS (25.0 months vs 26.6 months; *P* = .598). In particular, the PFS rates in the ENI and IFI groups were 58.9% and 64.4%, respectively, after 1 year (*P* = .440), 34.2% and 30.8% respectively after 3 years (*P* = .922), and 26.9% and 27.4%, respectively, after 5 years (*P* = .809, Figure 1). OS rates in the ENI and IFI groups were 84.6% and 82.5%, respectively, after 1 year (*P* = .628), 45.1% and 48.7%, respectively, after 3 years (*P* = .890) and 29.8% and 30.7%, respectively, after 5 years (*P* = .806, Figure 2).

**Figure 3:**
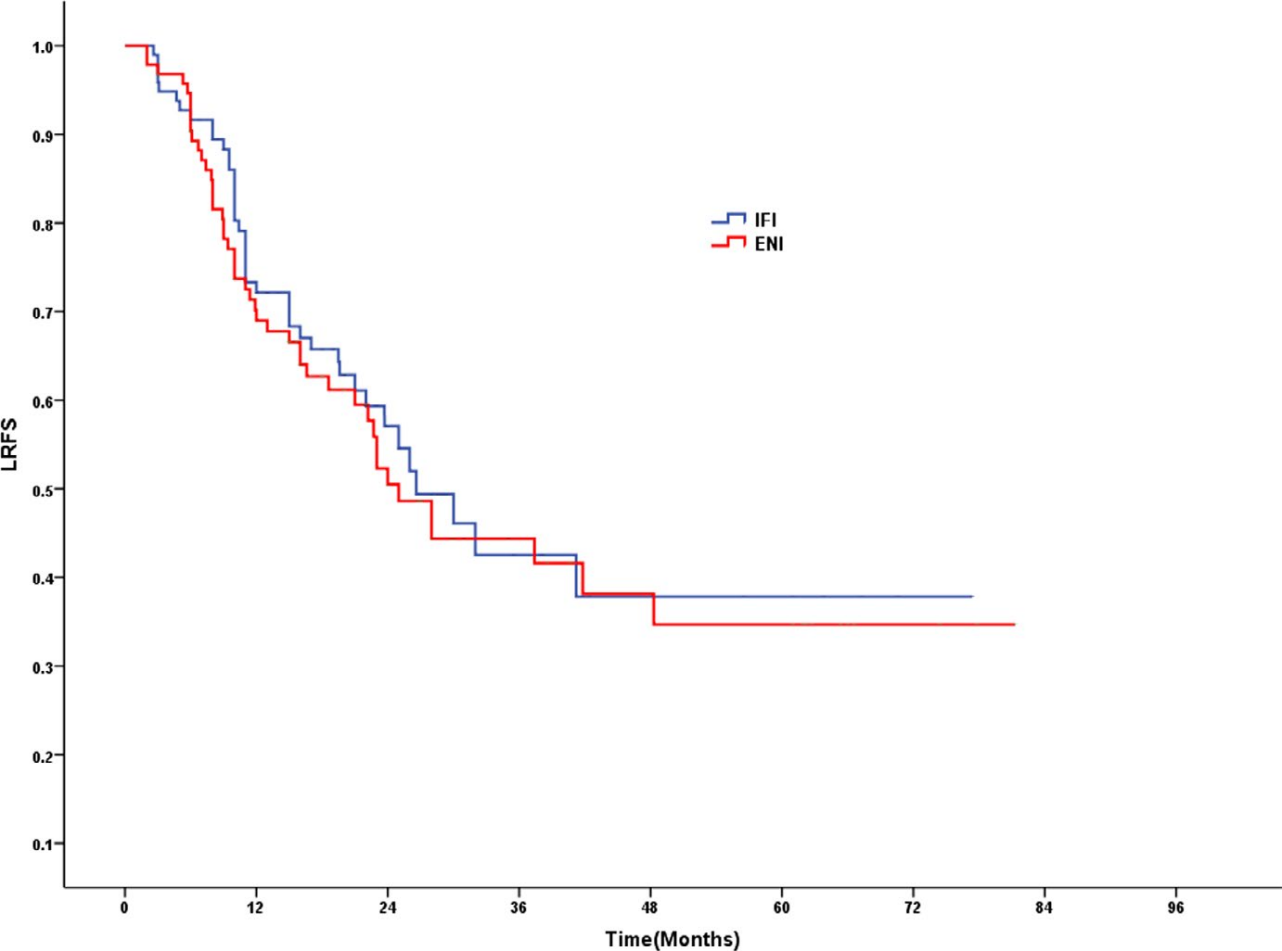
LRFS for patients in IFI group vs ENI group

**Figure 4:**
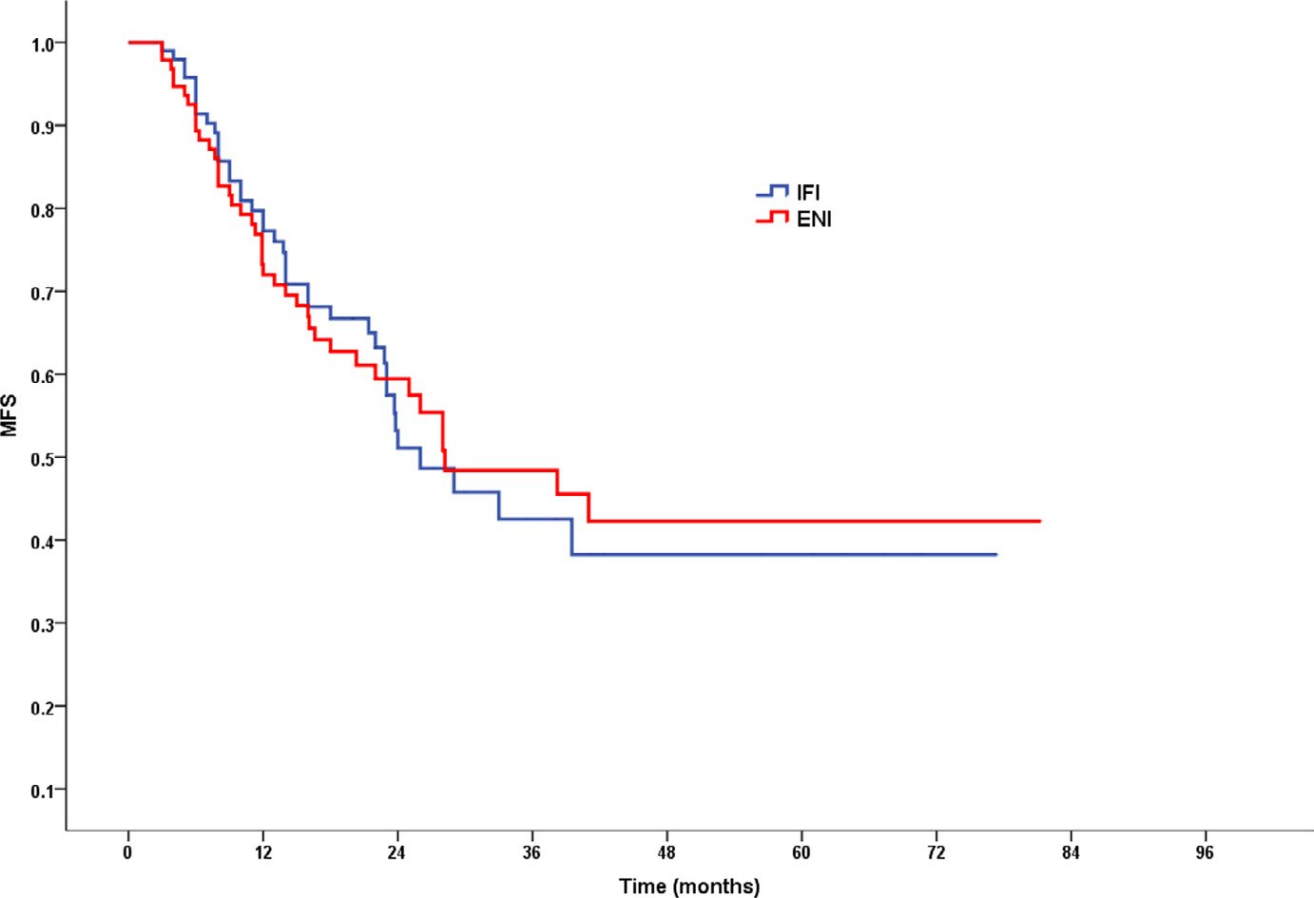
MFS for patients in IFI group vs ENI group

As of the last follow‐up, locoregional recurrence occurred in 41 patients (41.8%) in the IFI group and 46 patients (48.9%) in the ENI group (*P* = .385). No significant difference was found between the IFI group and the ENI group in the rate of metastasis (40.8% vs 43.6%, *P* = .694). After 1, 3, and 5 years, ENI patient LRFS rates were 72.1%, 42.5%, and 37.8%, respectively, compared with 69.0% (*P* = .537), 44.4% (*P* = .681), and 34.7% (*P* = .597), respectively, in the IFI group (Figure 3). Concomitantly, following 1, 3, and 5 years, ENI patient MFS rates were 77.3%, 42.5%, and 38.3%, respectively, vs respective IFI patient rates which were 72.0% (*P* = .418), 48.4% (*P* = .950), and 42.3% (*P* = .983) (Figure 4).

### 3.3 Univariate and multivariate analysis

Multivariate analysis suggested that OS was independently predicted by tumor response (*P* = .001) and clinical stage (*P* = .000) (Table 2). Meanwhile, PFS was independently influenced by the location of tumors, cStage, and tumor response (*P* = .044, 0.022, and 0.048, respectively) (Table 3).

**Table 2:** Univariate analysis demonstrating factors associated with OS

Subgroup	Univariate analysis		Multivariate analysis	
	HR (95% CI)	*P*	HR (95% CI)	*P*
Treatment group				
ENI	1.00		1.00	
IFI	0.985 (0.652‐1.488)	.941	0.950 (0.620‐1.454)	.812
Sex				
Female	1.00		1.00	
Male	0.858 (0.537‐1.370)	.520	0.789 (0.487‐1.278)	.336
Age, years				
≤60	1.00			
>60	0.886 (0.587‐1.337)	.563	0.928 (0.604‐1.427)	.734
KPS score				
80	1.00			
≥90	0.862 (0.567‐1.312)	.489	0.866 (0.562‐1.335)	.514
Tumor length (cm)				
>5	1.00			
≤5	0.837 (0.551‐1.273)	.406	0.879 (0.564‐1.371)	.570
Tumor location				
Ut	1.00			
Mt	1.132 (0.725‐1.766)	.586	1.347 (0.853‐2.126)	.201
Lt	1.569 (0.773‐3.185)	.212	1.082 (0.516‐2.268)	.834
cStage				
II	1.00			
III	3.919 (2.028‐7.573)	.000	3.996 (2.006‐7.960)	.000
Tumor response				
CR + PR	1.00			
SD + PD	2.688 (1.673‐4.320)	.000	2.335 (1.399‐3.898)	.001

Abbreviations: CR, complete response; cStage, clinical stage; ENI, elective nodal irradiation; IFI, involved‐field irradiation; Karnofsky Performance Status scores;KPS, Karnofsky performance status; Lt, lower thoracic; Mt, middle thoracic; PD, progressive disease; PR, partial response; SD, stable disease; Ut, upper thoracic.

**Table 3:** Univariate analysis demonstrating factors associated with PFS

Subgroup	Univariate analysis		Multivariate analysis	
	HR (95% CI)	*P*	HR (95% CI)	*P*
Treatment group				
ENI	1.00		1.00	
IFI	0.956 (0.661‐1.383)	.811	0.925 (0.628‐1.362)	.693
Sex				
Female	1.00		1.00	
Male	0.873 (0.579‐1.315)	.515	0.776 (0.511‐1.178)	.234
Age, years				
≤60	1.00			
>60	0.842 (0.582‐1.218)	.360	0.822 (0.561‐1.206)	.317
KPS score				
80	1.00			
≥90	0.900 (0.619‐1.309)	.582	0.834 (0.568‐1.225)	.356
Tumor length (cm)				
≤5	1.00			
>5	1.467 (1.011‐2.127)	.044	1.192 (0.795‐1.788)	.396
Tumor location				
Ut	1.00			
Mt	1.256 (0.847‐1.863)	.256	1.430 (0.955‐2.142)	.082
Lt	2.099 (1.076‐4.095)	.030	2.032 (1.019‐4.052)	.044
cStage				
II	1.00			
III	1.932 (1.220‐3.059)	.005	1.806 (1.089‐2.996)	.022
Tumor response				
CR + PR	1.00			
SD + PD	1.878 (1.155‐3.055)	.011	1.646 (1.005‐2.695)	.048

Abbreviations: CR, complete response; cStage, clinical stage; ENI, elective nodal irradiation; IFI, involved‐field irradiation; Karnofsky Performance Status scores;KPS, Karnofsky performance status; Lt, lower thoracic; Mt, middle thoracic; PD, progressive disease; PR, partial response; SD, stable disease; Ut, upper thoracic.

## 4 DISCUSSION

The results of Radiation Therapy Oncology Group 85‐01 study(3) led to CCRT becoming the standard treatment when EC patients refuse surgery or their tumors cannot be treated surgically. However, the optimal target volume of radiotherapy, especially the lymph node volume, remains controversial. ESCC has an extensive and longitudinal interconnecting lymphatic system in the esophageal wall.(7) It is conceivable that a specific dose delivered to areas surrounding noninvolved regional lymph nodes curb microscopic disease spread. Despite this, the necessity of ENI for EC has remained controversial because of its toxicity.

Acute esophagitis and pneumonitis are the main treatment‐related toxicities in EC patients receiving CCRT. Theoretically, a smaller CTV can reduce treatment‐related toxicity. In our primary results, we found that the incidences of grade ≥2 treatment‐related esophagitis and grade ≥2 acute treatment‐related pneumonitis were significantly lower in the IFI arm than in the ENI arm. Our findings demonstrated that IFI could reduce acute treatment‐related esophagitis and pneumonitis, which were confirmed by other published studies.(8, 9)

Despite its higher toxicity, some oncologists have also advocated using ENI because it may lead to better tumor local control and survival. A retrospective study using a propensity score matching method found that ENI is superior to IFI in improving OS in patients with ESCC. The median OS was 26.8 months for the ENI arm, vs 21.5 months for the IFI arm. ENI was a significant independent predictor of 5‐year OS (*P* = .015).(10) A meta‐analysis by Bai(11) found that extended‐field irradiation can reduce out‐of‐field failure rates in patients with EC.

However, whether ENI provides superior survival to IFI in patients with ESCC treated with radical radiotherapy remains controversial. Yamashita et al(12) found that ENI did not improve OS compared with IFI (IFI vs. ENI: 51.6% vs 34.8%, *P* = .087). In addition, more treatment‐related deaths were associated with ENI. In a study by Ma et al,(5) 102 patients with upper thoracic or cervical ESCC under IFI or ENI therapy were analyzed. ENI patients had a median survival time of 32.7 months which approximated closely to 33.7 months for IFI patients. There was no significant difference in the 3‐year survival rate between the ENI and IFI arms (41.3% vs 32.0%; *P* = .580). Cheng et al(13) conducted a meta‐analysis comparing ENI and IFI in treating ESCC to generate evidence to guide therapeutic decision making. They also found similarity in one, two, and three OS rates between IFI and ENI. Furthermore, a meta‐analysis of 757 patients with EC conducted by Wang et al(8) found no significant difference in OS between ENI and IFI patients.

To our knowledge, this is the first prospective, multicenter, randomized, controlled study comparing ENI and IFI in patients with EC. Our long‐term follow‐up results revealed that IFI was not associated with significant decreases in PFS, LRFS, MFS, and OS in patients with EC compared with ENI. Multivariate analysis further illustrated that the radiotherapy field (ENI and IFI) could not independently predict PFS or OS. Therefore, ENI was not superior to IFI for long‐term survival and tumor control. Consideration should therefore be given to IFI as an equivalent therapeutic alternative.

Few data have been reported to explain the mechanism by which IFI can achieve long‐term survival and tumor control similar to ENI. One potential reason may be incidental nodal irradiation. In thoracic EC, IFI can deliver a considerable incidental dose to elective regions, which could have significant effects on controlling micrometastases. Thirty‐nine patients who had untreatable thoracic ESCC had IFI administered through 3D conformal radiotherapy in study by Ji et al.(14) Under a 60.0‐Gy dosage, the median equivalent uniform dose was >40.0 Gy in most high‐risk nodal regions. Radiation doses of reduced intensity such as 24 Gy were shown to decrease local metastases by 30%–50% in lymphatic regions draining an EC affected site.(15) Contrarily, a large radiation volume (ENI) may reduce patient long‐term survival by negatively impacting the immune system.

In addition, all patients in our studies received concurrent chemotherapy. Chemotherapy has important significance for controlling lymph node micrometastasis and confers survival benefits in EC patients.(16, 17)

Furthermore, there is increasing evidence that radiotherapy effectively stimulates immune responses by increasing inflammatory cytokine production. This enhances immune exposure to tumor‐specific antigens thereby increasing immunological processing and presentation, improving T‐cell localization at disease sites and cytotoxic T‐cell mediated cancer cell destruction. Several clinical studies have also suggested that radiotherapy may enhance the comprehensive destruction of tumor cells by reinforcing the activity of the immune system.(18, 19, 20, 21)

Finally, as our previous report demonstrated, we posit that the broader ENI radiation fields cause greater injury, thereby reducing potential survival benefits.

To conclude, the survival rates of IFI‐treated patients were comparable to those of ENI‐treated patients. This provides evidence in support of the use of IFI in treating locally advanced EC while simultaneously lessening radiotoxicity.

## CONFLICT OF INTEREST

All authors disclose no conflict of interest.

## Data Availability

The data used to support the findings of this study are available from the corresponding author upon request.
